# Effects of schisandra extract on muscle atrophy: a systematic review and meta-analysis of preclinical studies

**DOI:** 10.3389/fphar.2026.1766559

**Published:** 2026-03-30

**Authors:** Xiao Liu, Chen Wang, Qi Song, Hai Liu, Youkang Zhu, Zhaochuan Xu, Xifang Liu

**Affiliations:** 1 Department of Rehabilitation, Honghui Hospital, Xi’an Jiaotong University, Xi’an, Shanxi, China; 2 School of Sports and Health Sciences, Xi’an Physical Education University, Xi’an, China

**Keywords:** animal models, meta-analysis, muscle atrophy, oxidative stress, schisandra

## Abstract

**Background:**

Lignans from *Schisandra chinensis*, such as schisandrin B, possess antioxidant, anti-inflammatory, and protein-regulating properties, suggesting potential against muscle atrophy. However, existing animal evidence is fragmented and lacks quantitative synthesis.

**Objective:**

This meta-analysis evaluates the effects of *S. chinensis* on various animal models of muscle atrophy, focusing on muscle structure, function, and oxidative stress.

**Methods:**

We systematically searched PubMed, Web of Science, Embase, Cochrane Library, and Ovid from inception to April 2025 for relevant animal RCTs, selected via the PICOS framework. Study quality was assessed using the SYRCLE tool. A random-effects meta-analysis was performed on outcomes including muscle weight, fiber cross-sectional area (CSA), catalase (CAT) activity, and grip strength.

**Results:**

Eleven studies (149 experimental, 149 control animals) were included. *S. chinensis* significantly increased muscle weight (standardized mean difference = 1.18, 95% CI: 0.19–2.16, P = 0.020) and CAT activity (SMD = 1.77, 95% CI: 0.31–3.23, P = 0.020). cross-sectional area improvement was borderline (SMD = 2.10, 95% CI: −0.02–4.22, P = 0.050). No significant changes occurred in body weight or grip strength. High heterogeneity (I^2^ = 80%–95%) was observed, attributable to variations in models and interventions.

**Conclusion:**

*S. chinensis* improves muscle structure potentially via enhanced antioxidant defense and reduced protein degradation, though functional benefits remain unconfirmed. Future high-quality, standardized studies are needed to clarify dose-response relationships and translational potential.

**Systematic Review Registration:**

identifier INPLASY202590030.

## 1 Introduction

Muscle atrophy is a well-characterized catabolic disorder characterized by a progressive loss of skeletal muscle mass and function. Its underlying mechanisms involve suppressed protein synthesis alongside increased protein breakdown, mediated primarily through the ubiquitin-proteasome system and the autophagy-lysosome pathway.

Additionally, mitochondrial dysfunction and sustained chronic inflammation are key contributors. Muscle atrophy severely impairs patients’quality of life and functional independence, and is commonly observed in various clinical conditions, including aging (leading to sarcopenia), chronic catabolic diseases (e.g., cancer cachexia), neurological injury (e.g., neurogenic atrophy), and disuse (e.g., prolonged bed rest) (Bodine and Baehr, 2014; Schiaffino et al., 2013). Globally, the prevalence of sarcopenia continues to rise, posing a major threat to human health. Epidemiological data indicate that this condition affects 10%–16% of older adults over 60 years of age worldwide, with prevalence exceeding 50% among those over 80 (Gao et al., 2021).

Given that no specific pharmacological therapy has yet been approved for sarcopenia, and existing agents (e.g., androgens, growth hormone, and myostatin inhibitors) show limited efficacy, their long-term safety risks (including immunogenicity, cardiovascular, and metabolic side effects) constrain their clinical prospects (Liu et al., 2025). While clinical interventions such as leucine, beta-hydroxy-beta-methylbutyrate (HMB), and resveratrol have shown promise in experimental muscle wasting, the multi-target profile of Schisandra lignans offers a unique combination of antioxidant and anti-proteolytic properties that warrants systematic investigation. Owing to their potent bioactive properties and a comparatively safe profile, phytochemicals have emerged as promising agents in the therapeutic intervention against metabolic syndrome, cancer, and cardiovascular diseases (Chen et al., 2024; Ayipo et al., 2024; El Oirdi, 2024; Elsori et al., 2024). Various natural compounds and nutritional supplements, such as leucine, β-hydroxy β-methylbutyrate (HMB), resveratrol, and urolithins, have been extensively studied for their potential to stimulate muscle protein synthesis or reduce systemic inflammation. While these agents offer specific benefits, the medicinal plant *Schisandra chinensis* has attracted particular attention due to its characteristic lignans (including schisandrin A, schisandrin B, and gomisin G) (Panossian and Wikman, 2008; Opletal et al., 2001) and its multi-target pharmacological activities, such as mitigating oxidative stress (Giridharan and Schisandrin, 2012; Chiu et al., 2011), alleviating inflammatory responses (Bian et al., 2022), and modulating signaling pathways (Bian et al., 2022). Based on these validated mechanisms, *S. chinensis* and its extracts have demonstrated therapeutic potential in hepatic protection (Addissouky et al., 2024), neuroprotection (He, 2014), and adjuvant cancer therapy (Jafernik et al., 2024). Furthermore, their relative safety as naturally derived compounds provide a strong rationale for investigating their role in sarcopenia interventions. Previous studies have shown that *S. chinensis* exerts beneficial effects in muscle atrophy models (Yeon et al., 2020), including enhancing antioxidant defense via activation of the Nrf2 pathway and reducing reactive oxygen species–mediated damage to muscle fibers (Wu et al., 2019; Li et al., 2025). However, these animal-derived findings remain fragmented and heterogeneous, involving diverse models, compounds, doses, and outcome measures.

In addition, although several reviews have discussed the therapeutic potential of *S. chinensis*, these narrative summaries lack quantitative synthesis and analysis of existing animal data, thereby limiting systematic evaluation of the overall effect, sources of heterogeneity, and strength of evidence. To bridge these evidence gaps (i.e., fragmented and heterogeneous findings, model limitations, and lack of quantitative synthesis), here, we performed a systematic review and meta-analysis of animal studies that investigated the effects of *S. chinensis* on diverse models of muscle atrophy (including aging, disuse, and drug-induced models). The objective of this study was to systematically evaluate the efficacy of *S. chinensis* and its lignans across a hierarchical range of outcomes in muscle atrophy models. Our primary research question focused on structural preservation (muscle weight and cross-sectional area [CSA]), while secondary objectives assessed functional recovery (grip strength) and the modulation of underlying molecular pathways (oxidative stress markers and proteolytic factors).

## 2 Methods

Adhering to the PRISMA 2020 statement, we designed and conducted this systematic review and meta-analysis, which had prior registration on INPLASY. Registration number:INPLASY202590030.

### 2.1 Search strategy

To identify relevant studies, we utilized a combination of Medical Subject Headings (MeSH) and free-text terms for key concepts related to the disease and intervention. A systematic literature search was performed across five electronic databases (PubMed, Web of Science, Embase, Cochrane Library, and Ovid full-text journals), encompassing all accessible records from their inception up to April 2025. No restrictions were applied regarding the publication language, including: i) Schisandra (“schizandra” OR “Gomisi” OR “Wuweizi”); ii) “muscle atrophy” (“muscular atrophy” OR “muscle wasting”). As an example, the detailed search strategy for PubMed is provided in Supplementary.

### 2.2 Inclusion and exclusion criteria

#### 2.2.1 Inclusion criteria

The inclusion criteria were established according to the PICOS framework: (i) Population: animal models of muscle atrophy; (ii) Intervention: treatment with Schisandra extracts (crude or standardized) or their major active lignan monomers (e.g., schisandrin A, schisandrin B); (iii) Outcomes: body weight and body composition indicators—body weight and muscle weight; muscle function—grip strength; histological and biochemical markersCSA of muscle fibers; protein expression—MuRF1, Atrogin1, and Myostatin; and serum/plasma biomarkers—malondialdehyde (MDA), superoxide dismutase (SOD), reactive oxygen species (ROS), CAT, and glutathione (GSH); (iv) Study design: RCTs.

#### 2.2.2 Exclusion criteria

Studies were excluded based on the following criteria: (i) non-relevant publication types, including *in vitro* studies, clinical trials, case reports, reviews, as well as editorials, abstracts, conference papers, and dissertations; (ii) studies in which Schisandra extracts were combined with other therapies; (iii) studies not involving muscle atrophy models; (iv) duplicate publications or studies with incomplete data; (v) studies without full-text availability; (vi) studies in which model groups received any additional pharmacological treatment; and (vii) studies without a model control group.

### 2.3 Data collection

We employed EndNote 20 to manage the literature obtained from the search. Following the removal of duplicates, two investigators independently performed the literature screening and data extraction based on pre-established inclusion and exclusion criteria (Qi et al., 2025). The initial screening of titles and abstracts led to the exclusion of irrelevant studies (Bian et al., 2025). Subsequently, the eligibility of the remaining articles was evaluated through a full-text review. All discrepancies between researchers were adjudicated by a third investigator until a consensus was achieved. The data extraction items included (Chen et al., 2021): (1) first author and publication year; (2) animal characteristics, including species, sex, body weight, and sample size; (3) anesthesia methodology; (4) modeling techniques and procedures; (5) intervention details (administration route, dosage, and treatment duration); (6) primary outcome measures. The following principles were applied to ensure consistent data handling: For studies featuring multiple dosage groups, a single group was selected for analysis. For consistency across studies, endpoint data were utilized in studies with serial measurements of outcomes. Data from studies using different modeling techniques were recorded separately. Among multiple publications from the same year, the one with the most complete data was chosen. Values presented graphically were extracted using Engauge Digitizer software.

### 2.4 Critical appraisal

Evaluation of methodological quality was performed according to the criteria of the SYRCLE tool (Hooijmans et al., 2014), which encompasses ten assessment domains (Javed and Romanos, 2025): (a) random sequence generation, (b) allocation concealment, (c) investigator blinding, (d) outcome assessor blinding, (e) random outcome assessment, (f) incomplete outcome data, (g) selective reporting, (h) baseline characteristics, (i) random housing conditions, and (j) other sources of bias. Every domain received a rating indicating a “low”, “high”, or “unclear” level of bias risk. A numerical score was assigned based on these ratings: 2 for “low,” 0 for “high,” and 1 for “unclear” risk. The maximum total score per study was 20 points.

### 2.5 Statistical analysis

RevMan 5.3 software was used to perform the meta-analysis of the extracted data. Effect sizes were expressed as standardized mean differences (SMDs) with corresponding 95% confidence intervals (95% CI). Homogeneity across studies was considered present when P ≥ 0.10 and I^2^ ≤ 50%, in which case a fixed-effects model was applied; otherwise, a random-effects model was used. Accordingly, a random-effects model was prespecified for this study. A significance level of 0.05 was adopted for all statistical analyses. To detect publication bias, funnel plots were constructed and subsequently analyzed using both Egger’s and Begg’s tests (Table 2).

## 3 Results

### 3.1 Study selection results

**FIGURE 1:**
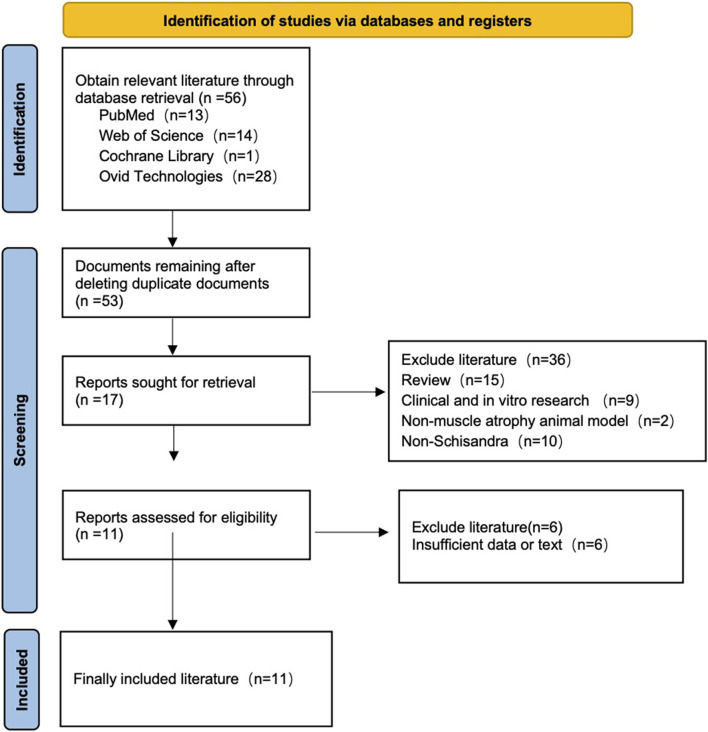
Flow diagram of study selection.

The preliminary screening of the five targeted databases resulted in the identification of 80 studies of potential relevance. After the removal of duplicates, 52 studies were identified as eligible for screening. Following a thorough evaluation of titles and abstracts, 12 studies were excluded. Consequently, 40 studies were selected for full-text review, of which 11 were ultimately included in the final analysis (Figure 1). The basic characteristics of the included studies are shown in Tables 1, 2.

**TABLE 1:** Basic characteristics of the included studies.

Animal characteristics	Model	Modeling material	Method of administration	Dose of schisandra	T	E	C	Outcome measures
Male C57BL/6 mice (4 weeks old)	Obesity	High-fat diet (HF)	Feed 60% calories as fat	0.25% OM(w/w)	56d	S	HF- induced obese mice were fed HF	①②③④⑤⑥⑦
Triploid crucian carp (17.13 ± 0.24 g)	Triploid crucian carp	​	​	​	63d	S	​	①②③⑧⑨⑩
C57BL/6 male mice (10 weeks old)	DEX	DMSO	Dissolved in 9% Kolliphor® HS 15 + 10% DMSO solution and orally administered 0.5% CMC	20 mg/kg SNA	10d	S	Injection of DEX dissolved in 9% Kolliphor® HS 15 + 10% DMSO solution and orally administered 0.5% CMC	①②③④⑤⑥⑦
C57BL/6N male mice (10 weeks old)	DMA	The spiral wire	Were immobilized using the spiral wire test to induce disuse muscle atrophy for 2 weeks	1 mg/kg gomisin G	14d	S	Received 9% Kolliphor® HS 15 + 10% DMSO orally	①②③④⑤⑥⑦
Adult SPF/VAF outbred CrljOri:CD1 (ICR) mice (35.76 ± 1.32 g)	DEX	Dexamethasone	Injection of dexamethasone (1 mg/kg), once a day for 10 days	250 mg/kg	24d	S	Water was orally	①②⑤⑥⑦⑧⑨⑩⑪⑫
Male C57BL/6 J mice (15 months old)	Non	Non	Non	0.1%Sfe(w/w)	120d	S	Fed a normal diet	①③④⑤⑥⑦
Adult SPF/VAF outbred CrljOri:CD1 (ICR) mice (29∼32 g)	NTX	Operation	Right sciatic neurectomy was performed by removing a 0.5 cm section of the nerve in the mid thigh	250 mg/kg	28d	S	Water was orally	①②⑤⑥⑦⑧⑨⑩⑪⑫
SPF/VAF male Hsd:ICR (CD-1) (32–54 g)	Non	Non	Non	250 mg/kg	28d	S	Orally administered distilled water	②⑤⑥⑦⑧⑨⑩⑪⑫
Male sprague-dawley (SD) rats (210–240 g)	DMA	Cast	Cast immobilization	20 mg/kg	21d	S	Oral saline	①②③④
Male C57BL/6 mice (8 weeks old)	STZ-diabetic	STZ	Intraperitoneally injected	250 mg/kg	42d	S	Orally administered 9% Kolliphor® HS 15 + 10% DMSO	①②③④⑤⑥⑦
Female SD rats (10 weeks old)	OVX	​	​	10 mg/kg	56d	S	Adminis- tered orally	①②

*SNA*, schisandrin A; *OM*, oil macerate; *Sfe*, supercritical fluid extract; *DEX*, dexamethasone; *NTX*, neurectomy; *DMA*, disuse muscle atrophy.

**TABLE 2:** Meta-analysis results for each outcome indicator.

Outcome indictor	Heterogeneity test results		Effect models	Meta-analysis results		
​	I^2^ (%)	P	​	Effect sizes	95%CI	P
Body weight	91	<0.001	Random	SMD = 0.30	[-0.95, 1.55]	0.640
Muscle weight	85	<0.001	Random	SMD = 1.18	[0.19, 2.16]	0.020
CSA	82	<0.001	Random	SMD = 2.10	[-0.02, 4.22]	0.050
Grip strength	95	<0.001	Random	SMD = 1.38	[-1.18, 3.93]	0.290
Myostatin	89	<0.001	Random	SMD = −2.75	[-4.23, -1.27]	<0.001
MuRF1	91	<0.001	Random	SMD = −3.20	[-4.99, -1.40]	<0.001
Atrogin1	89	<0.001	Random	SMD = −2.58	[-3.97, -1.20]	<0.001
SOD	91	<0.001	Random	SMD = 2.34	[-0.07, 4.76]	0.060
MDA	85	<0.001	Random	SMD = −1.66	[-3.34, 0.02]	0.050
CAT	80	0.002	Random	SMD = 1.77	[0.31, 3.23]	0.020
ROS	82	0.004	Random	SMD = −0.82	[-2.28, 0.65]	0.270
GSH	85	0.001	Random	SMD = 1.19	[-0.50, 2.88]	0.170

### 3.2 Summary of included studies

Eleven studies met the pre-defined inclusion criteria. A total of three animal types were involved across the included publications: mice (8 studies), rats (2 studies), and fish (1 study). A model control group was an integral component of the experimental design in all studies. The specific control types were distributed as follows: vehicle control (n = 7), untreated control (n = 3), and sham-operated control (n = 1). Notably, various routes of administration were employed across the studies. Regarding compound delivery, oral and intraperitoneal routes were employed in ten and one of the studies, respectively. The key features of all included studies are tabulated in the Supplementary Materials.

### 3.3 Quality assessment of included studies

Due to the consistent reporting of controlled housing conditions (including temperature, humidity, and light-dark cycles) across all studies, the fundamental criterion for random housing was considered met. This domain was accordingly assigned a rating of “low” or “unclear” risk of bias. The total quality ratings for the included investigations varied between 9 and 14 points.

Baseline characteristics were reported as not significantly different between groups in all studies, and thus this domain was rated as “low risk.” No investigation explicitly described the methods of randomization; 4 merely mentioned “random grouping” and were rated as “unclear risk,” whereas 7 provided no randomization details and were rated as “high risk.” Allocation concealment was not reported in any study, resulting in ratings of “high risk” or “unclear risk.” None of the studies reported whether outcome assessment was randomized, and thus this domain was rated as “unclear risk.” The blinding procedures for both investigators and outcome assessors were inadequately described in all studies. Six trials were rated as “high risk” owing to the likely identifiability of the treatment assignments. Whereas the remaining five were designated as “unclear risk” because of limited methodological details. All studies demonstrated complete outcome reporting with no exclusions, resulting in a “low risk” rating in this domain. No indication of selective reporting was detected across the studies, and all were assessed as “low risk” in this domain. None of the 11 studies reported apparent commercial sponsorship or conflicts of interest, and all were rated as “low risk.” Collectively, the methodological rigor of the encompassed investigations was judged to be intermediate, with a tendency toward the lower end. Several shortcomings were identified in the design and implementation of in vivostudies; addressing these issues is critical to strengthen the validity of preclinical results and facilitate their clinical translation. A full breakdown of the quality assessment is provided in Table 3.

**TABLE 3:** Quality and risk of bias assessment.

Study	a	b	c	d	e	f	g	h	i	j	Score
Yoo et al. (2022)	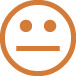	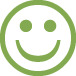	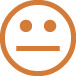	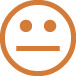	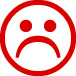	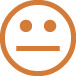	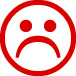	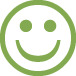	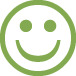	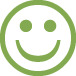	12
Zuo et al. (2023)	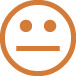	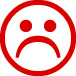	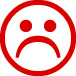	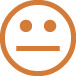	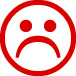	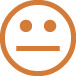	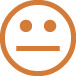	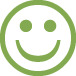	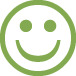	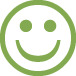	10
Yeon et al. (2020)	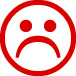	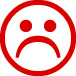	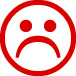	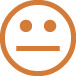	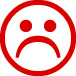	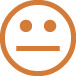	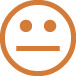	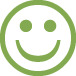	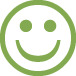	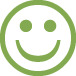	9
Yeon et al. (2022)	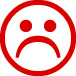	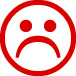	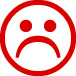	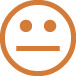	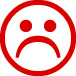	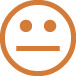	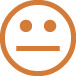	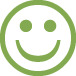	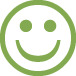	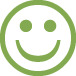	9
Kim et al. (2015a)	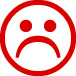	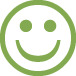	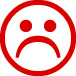	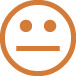	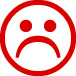	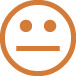	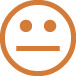	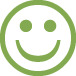	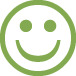	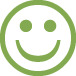	10
Choi et al. (2019)	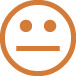	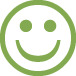	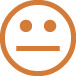	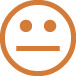	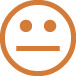	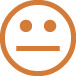	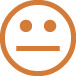	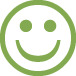	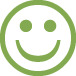	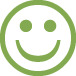	13
Kim et al. (2015b)	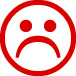	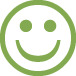	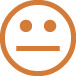	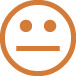	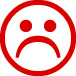	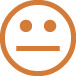	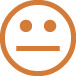	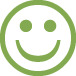	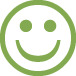	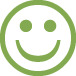	12
Kim et al. (2018)	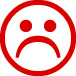	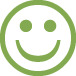	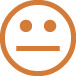	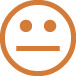	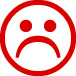	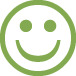	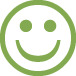	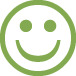	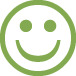	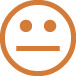	11
Cho et al. (2018)	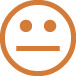	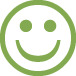	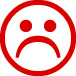	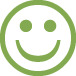	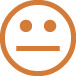	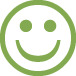	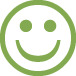	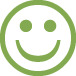	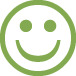	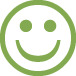	14
Choi et al. (2021)	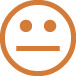	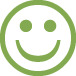	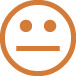	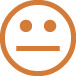	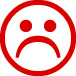	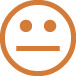	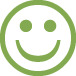	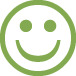	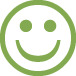	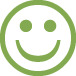	14
Kim et al. (2019)	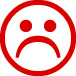	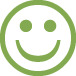	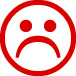	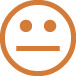	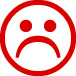	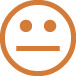	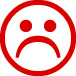	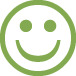	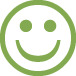	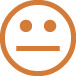	9

### 3.4 Meta-analytic results

#### 3.4.1 Regulation of oxidative stress markers

**FIGURE 2:**
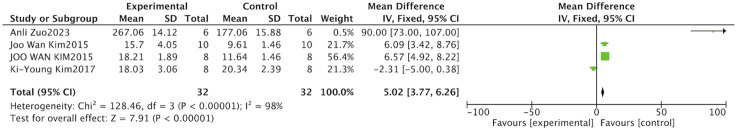
Forest plot of superoxide dismutase (SOD) activity. The plot displays the standardized mean difference (SMD) with 95% confidence intervals (CIs). A random-effects model was employed due to high heterogeneity (I^2^ = 91%). Data points to the right of the vertical zero line indicate increased SOD activity in the *Schisandra*-treated group.

SOD is a key antioxidant enzyme whose primary function is to eliminate superoxide radicals in the body and thereby mitigate oxidative damage (Zheng et al., 2023; Chen et al., 2023). Muscle atrophy is typically associated with elevated oxidative stress (Jang et al., 2020; Hu et al., 2022a). Three studies investigated the effects of Schisandra on serum SOD activity, and owing to considerable heterogeneity across the studies, a random-effects model was employed to perform the meta-analysis (I^2^ = 91%, P < 0.001). The results indicated that serum SOD activity was higher in the Schisandra-treated groups compared with model controls, although the effect did not reach statistical significance (SMD = 2.34, 95% CI: [-0.07, 4.76], P = 0.060) (Figure 2).

**FIGURE 3:**
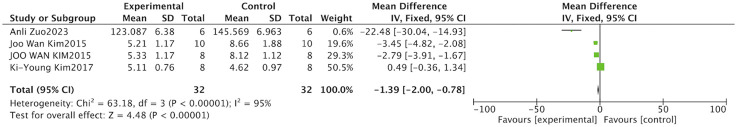
Forest plot of malondialdehyde (MDA) concentrations. The plot evaluates lipid peroxidation markers using SMD and 95% CI. Results show a borderline reduction in the treatment group (*P =* 0.050). A random-effects model was used (I^2^ = 85%).

MDA represents a primary byproduct generated through the process of lipid peroxidation. Owing to its ability to form covalent bonds with biological macromolecules including proteins and DNA, MDA promotes adduct formation (Tian et al., 2025). This process leads to protein destabilization and genomic injury, ultimately aggravating cellular damage (Zhao et al., 2024). A total cohort of four studies evaluated the impact of *S. chinensis* on serum concentrations of MDA. A random-effects model was applied for the meta-analysis due to considerable heterogeneity (I^2^ = 85%, P < 0.001). The results indicated that serum MDA levels were lower in the Schisandra-treated groups compared with controls, with a trend toward significant difference (SMD = −1.66, 95% CI: [-3.34, 0.02], P = 0.050) (Figure 3).

**FIGURE 4:**
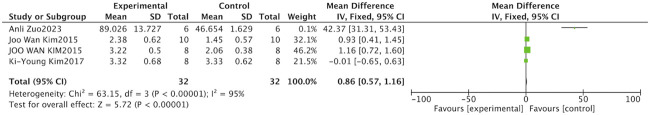
Forest plot of catalase (CAT) activity. This analysis demonstrates a significant elevation of CAT activity following *Schisandra* intervention (*P =* 0.020). Heterogeneity was moderate (I^2^ = 80%), and a random-effects model was applied.

As a key antioxidant enzyme, CAT facilitates the breakdown of hydrogen peroxide into water and oxygen, mitigating damage caused by oxidative stress (Patil et al., 2006). Four studies examined the effects of Schisandra on serum CAT activity. Given the significant heterogeneity observed across studies, a random-effects approach was utilized in the meta-analysis (I^2^ = 80%, P = 0.002). Data demonstrated a marked elevation in catalase (CAT) activity within the serum of animals receiving Schisandra treatment, relative to model control subjects (SMD = 1.77, 95% CI: [0.31, 3.23], P = 0.020) (Figure 4).

**FIGURE 5:**
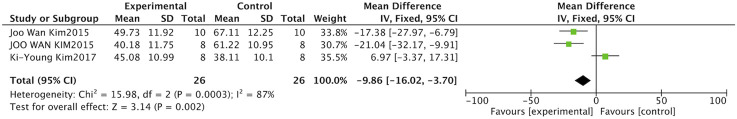
Forest plot of reactive oxygen species (ROS) levels. The forest plot synthesizes the impact of *Schisandra* on systemic ROS production. No statistically significant difference was observed (*P =* 0.270).

ROS are highly reactive oxygen-containing molecules. When ROS production exceeds the body’s clearance capacity, oxidative stress occurs, damaging cells and tissues and contributing to the development of various diseases (Jang et al., 2020). The influence of Schisandra on serum ROS activity was evaluated in three studies. The meta-analysis employed a random-effects approach on account of marked heterogeneity observed across the included studies (I^2^ = 91%, P < 0.001). Analysis revealed reduced serum ROS levels in Schisandra-treated groups relative to model controls, although this difference did not reach statistical significance. (SMD = –0.82, 95% CI: [-2.28,0.65], P = 0.270) (Figure 5).

**FIGURE 6:**
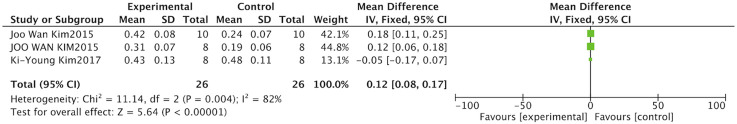
Forest plot of glutathione (GSH) activity. The analysis compares GSH levels between *Schisandra* and model control groups. The results show a non-significant trend toward increased GSH (*P =* 0.170).

GSH is a non-enzymatic antioxidant that participates in the elimination of ROS, maintenance of redox balance, and detoxification processes (Kondo et al., 1993). Three investigations evaluated the influence of Schisandra on GSH activity in serum. Given the considerable variability observed across studies, the meta-analysis was conducted using a random-effects approach (I^2^ = 85%, P = 0.001). The results indicated that serum GSH activity was higher in the Schisandra-treated groups compared with model controls, but the observed effect did not reach the threshold for statistical significance. (SMD = 1.19, 95% CI: [-0.50,2.88], P = 0.170) (Figure 6).

#### 3.4.2 Body weight

**FIGURE 7:**
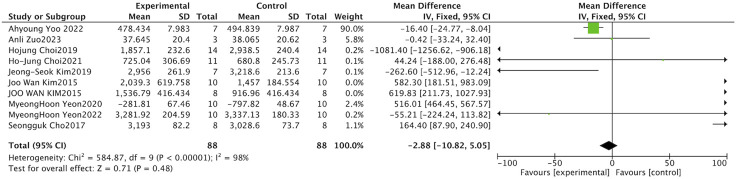
Forest plot of total body weight. The combined data indicate that *Schisandra* intervention did not significantly affect overall body mass (*P =* 0.640), suggesting the effects are muscle-specific rather than systemic.

Body weight is a fundamental indicator of nutritional and metabolic status and serves as an important monitoring parameter in disease models related to muscle atrophy. The meta-analysis of ten studies examining the impact of *S. chinensis* on body weight utilized a random-effects approach, as considerable heterogeneity was detected among the included investigations (I^2^ = 91%, P < 0.001). The combined data from the meta-analysis revealed no statistically significant alteration in body mass compared to model control groups (SMD = 0.30, 95% CI: 0.95 to 1.55; Z = 0.47, *P =* 0.640). The confidence interval crossed zero and the P-value exceeded 0.05, confirming the absence of statistical significance (Figure 7).

#### 3.4.3 Muscle weight

**FIGURE 8:**
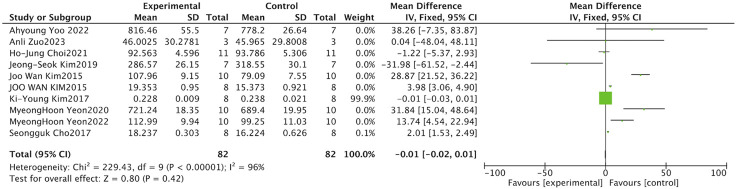
Forest plot of muscle weight. This primary structural outcome demonstrates that *Schisandra* significantly increases muscle weight compared to model controls (*P =* 0.020). The diamond represents the pooled SMD estimate.

Muscle weight is a direct pathological indicator for evaluating the degree of muscle atrophy and holds important diagnostic value in studies of neuromuscular disorders and metabolic dysregulation. Nine studies examined the effects of *S. chinensis* on muscle weight. Owing to substantial heterogeneity, a random-effects model was employed for the meta-analysis. (I^2^ = 85%, P < 0.001). The results indicated that Schisandra intervention significantly increased muscle weight compared with model controls (pooled SMD = 1.18, 95% CI: [0.19,2.16], Z = 2.35, P = 0.020). The confidence interval did not cross zero and the P-value was <0.05, confirming statistical significance (Figure 8).

#### 3.4.4 CSA

**FIGURE 9:**
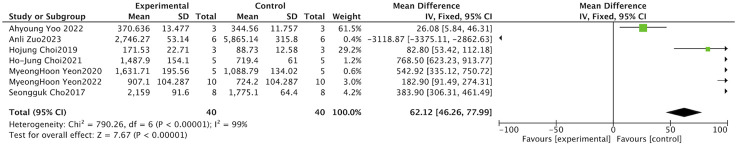
Forest plot of fiber cross-sectional area (CSA). Morphometric analysis shows an improvement trend in myofiber size at the threshold of significance (*P =* 0.050). The analysis accounts for substantial heterogeneity across diverse atrophy models.

CSA of muscle fibers is a core morphological indicator for evaluating pathological changes in muscle atrophy and holds important diagnostic value in neuromuscular disease models (Hu et al., 2022a). The effects of Schisandra on CSA were examined in seven studies. Given the considerable heterogeneity identified, the meta-analysis utilized a random-effects approach (I^2^ = 82%, P < 0.001). The results indicated that Schisandra intervention produced an improvement in CSA that was at the threshold of statistical significance (pooled SMD = 2.10, 95% CI: [-0.02, 4.22], Z = 1.94, P = 0.050). The lower bound of the confidence interval approached zero, suggesting borderline significance (Figure 9).

#### 3.4.5 Grip strength

**FIGURE 10:**
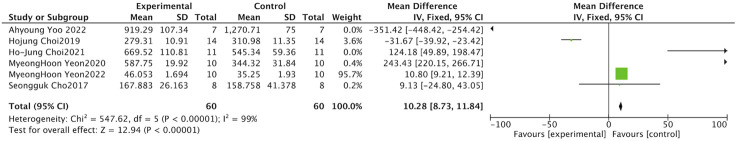
Forest plot of functional grip strength. The plot indicates no statistically significant improvement in grip strength recovery (*P =* 0.290), highlighting a structural-functional dissociation.

Grip strength is a core indicator for evaluating muscle function and has important clinical relevance in studies of neuromuscular disorders and aging. Owing to substantial heterogeneity, for the meta-analysis examining the impact of Schisandra on grip strength across six studies, a random-effects modeling approach was applied (I^2^ = 95%, P < 0.001). Results showed that Schisandra administration did not demonstrate a significant improvement in grip strength relative to model controls. (pooled SMD = 1.38, 95% CI: [-1.18, 3.93], Z = 1.05, P = 0.290). The confidence interval encompassed the value of zero, accompanied by a P-value above the 0.05 threshold, which together indicate a lack of statistical significance (Figure 10).

#### 3.4.6 Core regulatory factors of muscle atrophy

**FIGURE 11:**
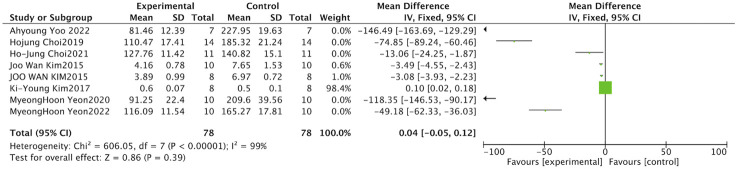
Forest plot of Myostatin expression. This mechanistic outcome illustrates a significant suppression of Myostatin, a negative regulator of muscle growth, in the treatment group (P < 0.001).

Myostatin is a negative regulator of muscle growth that restricts hypertrophy by inhibiting muscle protein synthesis and satellite cell activation (Bodine and Baehr, 2014; Hu et al., 2022a). A total of 8 independent studies evaluated the effects of Schisandra on Myostatin expression. High heterogeneity was observed across studies (I^2^ = 89%, P < 0.001), and therefore a random-effects model was applied for the meta-analysis. The results indicated that Schisandra intervention significantly reduced Myostatin expression compared with model controls (SMD = –2.75, 95% CI: [-42.3, −1.27]). This effect was highly statistically significant (Z = 3.64, P < 0.001), with the confidence interval entirely in the negative range and not crossing zero. These findings suggest that Schisandra may alleviate the negative feedback on muscle growth by effectively suppressing Myostatin overexpression (Figure 11).

**FIGURE 12:**
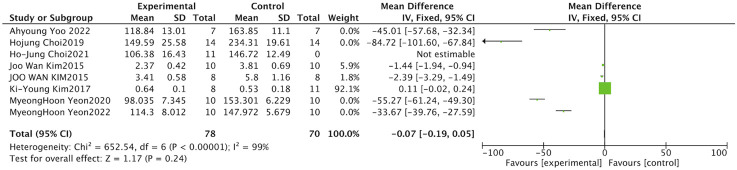
Forest plot of MuRF1 expression. The analysis demonstrates that *Schisandra* significantly downregulates MuRF1, a key E3 ubiquitin ligase involved in protein degradation (P < 0.001).

MuRF1 is a key effector molecule in muscle protein degradation (Bodine and Baehr, 2014). A series of seven independent investigations evaluated the influence of Schisandra on MuRF1 expression. The presence of considerable heterogeneity (I^2^ = 91%, P < 0.001) justified the use of a random-effects model for the analysis (Yu et al., 2023). The results indicated that Schisandra intervention significantly reduced MuRF1 expression relative to model control groups (SMD = –3.20, 95% CI: [-4.99, −1.40]). This inhibitory effect was highly statistically significant (Z = 3.49, P < 0.001), with the confidence interval entirely in the negative range and not crossing zero. These findings suggest that Schisandra may counteract muscle atrophy by suppressing MuRF1 expression, thereby reducing ubiquitination and degradation of contractile proteins (Figure 12).

**FIGURE 13:**
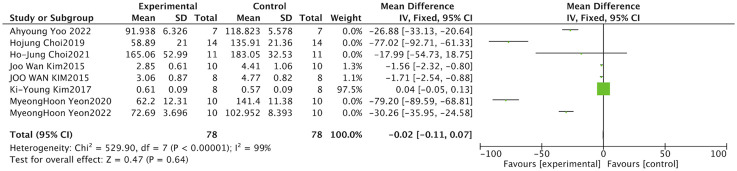
Forest plot of Atrogin-1 expression. Similar to MuRF1, *Schisandra* significantly inhibits the expression of Atrogin-1 (P < 0.001), confirming its role in suppressing the ubiquitin-proteasome proteolytic pathway.

Atrogin-1 participates in muscle protein degradation through the ubiquitin–proteasome pathway and is considered one of the core biomarkers of muscle atrophy (Bodine and Baehr, 2014; Schiaffino et al., 2013) Eight studies examined the influence of Schisandra on Atrogin-1 expression. High heterogeneity was observed across studies (Tau^2^ = 3.41; Chi^2^ = 64.73, df = 7, P < 0.001; I^2^ = 89%), a and a random-effects model was accordingly adopted. The results indicated that Schisandra intervention significantly reduced Atrogin-1 expression compared with model controls (pooled SMD = –2.58, 95% CI: [-3.97, −1.20]). This effect was highly statistically significant (Z = 3.66, P < 0.001), with the confidence interval entirely within the negative range and not crossing zero. This finding was highly consistent with the results for MuRF1, clearly suggesting that Schisandra can effectively suppress activation of the ubiquitin–proteasome pathway (Figure 13).

#### 3.4.7 Meta-regression analyses of dose and intervention duration

To further explore potential sources of heterogeneity, random-effects meta-regression analyses were conducted to examine the influence of dose and intervention duration on outcome effect sizes (Table 4). Overall, no significant dose-dependent associations were observed for body weight, muscle weight, CSA, grip strength, or most molecular and oxidative stress–related markers (all p > 0.05).

**TABLE 4:** Meta-regression analyses of dose and intervention duration on outcome effect sizes.

Outcome	Factor	Coef	Std. Err	z	P > |z|	[95%Conf]	[Interval]
Body weight	Dose	−0.0074275	0.0062612	−1.19	0.236	−0.0196993	0.0048443
Body weight	Duration	0.0053471	0.056282	0.01	0.924	−0.1049636	0.1156577
Muscle weight	Dose	0.0009144	0.0064069	0.14	0.887	−0.0116428	0.0134716
Muscle weight	Duration	−0.0746966	0.0433076	−1.72	0.085	−0.1595779	0.0101848
CSA	Dose	0.0126823	0.0103525	1.23	0.221	−0.0076083	0.0329729
CSA	Duration	0.1585068	0.0544632	2.91	0.004	0.0517609	0.2652528
Grip strength	Dose	−0.0146687	0.0204096	−0.72	0.472	−0.0546707	0.0253334
Grip strength	Duration	−0.1881987	0.1388243	−1.36	0.175	−0.4602893	0.0838919
Myostatin	Dose	0.0079454	0.006402	1.24	0.215	−0.0046023	0.0204931
Myostatin	Duration	0.0936602	0.0658752	1.42	0.155	−0.0354529	0.2227733
MuRF1	Dose	0.0175088	0.0069998	2.50	0.012	0.0037895	0.0312282
MuRF1	Duration	0.1512983	0.0949614	1.59	0.111	−0.0348227	0.3374192
Atrogin1	Dose	0.0175088	0.0069998	2.50	0.012	0.0037895	0.0312282
Atrogin1	Duration	0.1512983	0.0949614	1.59	0.111	−0.0348227	0.3374192
SOD	Dose	-	-	-	-	-	-
SOD	Duration	−0.7811845	0.6253428	−1.25	0.212	−2.006834	0.4444649
MDA	Dose	-	-	-	-	-	-
MDA	Duration	0.382064	0.5855054	0.65	0.514	−0.7655056	1.529634
CAT	Dose	-	-	-	-	-	-
CAT	Duration	−0.4338195	0.3583474	−1.21	0.226	−1.136168	0.2685284
ROS	Dose	-	-	-	-	-	-
ROS	Duration	0.3433395	0.4453707	0.77	0.441	−0.5295711	1.21625
GSH	Dose	-	-	-	-	-	-
GSH	Duration	−0.2024312	0.5867137	−0.35	0.730	−1.352369	0.9475066

With respect to intervention duration, a significant positive association was observed for muscle fiber cross-sectional area (CSA) (β = 0.159, p = 0.004), indicating larger effect sizes with longer treatment periods. For other outcomes, including body weight, muscle weight, grip strength, and molecular markers, intervention duration was not significantly associated with effect size (all p > 0.05). Dose could not be evaluated for several oxidative stress–related outcomes due to differences in dosing units across studies. Notably, while *S. chinensis* significantly improved muscle structural markers such as muscle weight, these changes did not translate into statistically significant improvements in functional grip strength (SMD = 1.38, P = 0.29). This suggests a potential lag or dissociation between morphometric recovery and functional performance in the included animal models.

## 4 Discussion

### 4.1 Synthesis of findings

A meta-analysis was conducted on aggregated data derived from eleven preclinical investigations, which confirmed that Schisandra extracts ameliorated muscle structure in atrophy models primarily by modulating key molecular pathways and oxidative stress; however, the effects varied significantly across different outcome measures. Structurally, Schisandra significantly increased muscle weight (SMD = 1.18, 95% CI: [0.19, 2.16], P = 0.020) and showed borderline significant improvement in CSA (SMD = 2.10, 95% CI: [-0.02, 4.22], P = 0.050), suggesting a protective effect on muscle morphology. However, functional outcomes, including grip strength, did not demonstrate statistical significance (SMD = 1.38, 95% CI: [-1.18, 3.93], P = 0.290), indicating insufficient evidence for functional recovery. At the molecular level, Schisandra exerted potent inhibitory effects on protein degradation pathways, including significant reductions in Myostatin expression (SMD = –2.75, 95% CI: [-42.3, −1.27], P < 0.001), MuRF1 expression (SMD = –3.20, 95% CI: [-4.99, −1.40], P < 0.001), and Atrogin-1 expression (SMD = –2.58, 95% CI: [-3.97, −1.20], P < 0.001). These findings highlight the suppression of the ubiquitin-proteasome pathway as a central mechanism by which Schisandra alleviates muscle atrophy (Wang et al., 2022).

With respect to oxidative stress regulation, Schisandra demonstrated beneficial effects on certain antioxidant markers, as seen in the substantial upregulation of CAT activity (SMD = 1.77, 95% CI: [0.31, 3.23], P = 0.020). However, improvements in other markers—including SOD activity (SMD = 2.34, P = 0.06), GSH activity (SMD = 1.19, P = 0.17), and ROS levels (SMD = –0.82, P = 0.27),did not reach statistical significance. MDA levels showed only a borderline reduction (SMD = –1.66, 95% CI: [-3.34, 0.02], P = 0.05). Notably, body weight was entirely unaffected (SMD = 0.30, P = 0.640), suggesting that the effects of Schisandra were specific to muscle tissue rather than systemic metabolism (Hu et al., 2022b).

However, these findings are constrained by substantial heterogeneity and methodological shortcomings. All indicators exhibited considerable heterogeneity (I^2^ = 80%–95%), primarily attributable to variability in animal models, differences in modeling methods, and inconsistencies in intervention protocols. Because the included studies had relatively small sample sizes, subgroup analyses to accurately quantify the sources of heterogeneity were not feasible, and the absence of sensitivity analyses further limited the assessment of result stability. The methodological rigor of the studies was evaluated as low to moderate overall, as indicated by quality scores of merely 9 to 14 (maximum score: 20). Specifically, randomization methods were unclear (seven studies did not report details and were considered high risk; three studies grouped animals solely by weight, also representing a high risk of bias). None of the studies implemented allocation concealment or blinded assessment (six were judged high risk due to identifiable interventions, while five were rated as unclear risk due to insufficient reporting), substantially increasing the likelihood of bias. The dose–response relationship also remains unclear, as different studies employed inconsistent dosages and treatment durations, which warrants prioritized investigation aimed at establishing the optimal therapeutic window (Author anonymus, 2025).

In summary, Schisandra effectively alleviates muscle atrophy by suppressing atrophy-related genes (Myostatin, MuRF1, Atrogin1) and enhancing specific antioxidant defenses (e.g., CAT). However, the evidence remains weak regarding functional outcomes and certain oxidative stress markers. Therefore, we strongly recommend that future studies prioritize improving methodological quality by employing computer-generated randomization, rigorously implementing blinding and allocation concealment, and conducting dose-response investigations in diverse muscle atrophy models (such as cancer cachexia and sarcopenia) to establish a more reliable foundation for clinical translation.

### 4.2 Effectiveness

The pathological progression of muscle atrophy is closely associated with oxidative stress–induced damage. Inhibition of protein synthesis (Schiaffino et al., 2013), mitochondrial dysfunction, and chronic inflammatory responses can lead to excessive accumulation of ROS and reactive nitrogen species (RNS) (Zarkovic, 2020), triggering lipid peroxidation and protein oxidation, and ultimately accelerating muscle fiber degradation. Among these, MDA, a key end-product of lipid peroxidation, serves as a direct marker of oxidative damage when its levels are elevated (Huang et al., 2024). The present meta-analysis confirmed that the antioxidant activity of Schisandra lignans (e.g.,.schisandrin A and schisandrin B) is primarily mediated through two key pathways: direct free radical scavenging and activation of endogenous antioxidant defenses. Specifically, Schisandra significantly increased CAT activity (SMD = 1.77, 95% CI [0.31, 3.23], P = 0.02) and marginally reduced MDA levels (SMD = −1.66, 95% CI [-3.34, 0.02], P = 0.05), thereby confirming its efficacy against lipid peroxidation. However, the increase in SOD activity did not reach statistical significance (SMD = 2.34, P = 0.06), and no significant changes were observed in ROS (SMD = −0.82, P = 0.27) or GSH (SMD = 1.19, P = 0.17). These findings suggest that its antioxidant effects are selectively targeted, preferentially enhancing CAT-mediated hydrogen peroxide decomposition rather than SOD-related superoxide clearance.

Multiple experimental studies have confirmed that the muscle-protective effects of Schisandra are directly associated with the regulation of molecular signaling pathways. For example, Yeon et al. demonstrated that schisandrin A attenuated dexamethasone-induced muscle atrophy by suppressing the expression of key ubiquitin–proteasome pathway factors, MuRF1 and Atrogin-1, with reductions of 58%–62% (Yeon et al., 2020). These findings are highly consistent with the present meta-analysis, which showed a significant decrease in MuRF1 expression (SMD = −3.20, 95% CI [-4.99, −1.40], P = 0.0005) and a parallel suppression of Atrogin-1 expression (SMD = −2.58, 95% CI [-3.97, −1.20], P = 0.0003). Furthermore, Wu et al. revealed that Schisandra enhances antioxidant defenses by activating the Nrf2 pathway (Wu et al., 2019; Li et al., 2025), a finding consistent with the observed upregulation of CAT activity. Of particular significance is the observed suppression of key inflammatory cytokines, including TNF-α and IL-6, by Schisandra lignans (Bian et al., 2022), thereby interrupting the vicious cycle between oxidative stress and inflammation. This establishes a multi-target protective mechanism characterized by three synergistic effects: mitigation of oxidative stress (CAT↑/MDA↓), inhibition of inflammation (downregulation of pro-inflammatory cytokines), and regulation of protein metabolism (MuRF1/Atrogin1↓), collectively attenuating the progression of muscle atrophy.

In terms of structural muscle repair, Schisandra significantly increased muscle weight (SMD = 1.18, 95% CI [0.19, 2.16], P = 0.02), a finding directly linked to the mechanism described by Yeon et al. (2020), whereby suppression of MuRF1/Atrogin-1 expression effectively prevents muscle fiber degradation. Meanwhile, the CSA of muscle fibers showed a borderline improvement trend (SMD = 2.10, 95% CI [-0.02, 4.22], P = 0.05), suggesting a potential protective effect on muscle fiber morphology. However, this structural repair did not fully translate into systemic functional improvements: body weight changes were not reliably different (SMD = 0.30, 95% CI [-0.95, 1.55], P = 0.64), confirming that the intervention specifically targeted muscle tissue without affecting overall metabolism. Grip strength also did not improve significantly (SMD = 1.38, 95% CI [-1.18, 3.93], P = 0.29), reflecting that neuromuscular coordination recovery requires more comprehensive strategies. This structural–functional dissociation highlights the core value of Schisandra in halting the pathological progression of atrophy, while functional reconstruction may require combination with exercise or neuromodulatory therapies to achieve clinical translation. In summary, evidence from 11 preclinical studies indicates that Schisandra alleviates structural damage in muscle atrophy through multi-target mechanisms involving antioxidation, anti-inflammation, and anti-protein degradation. However, the lack of functional recovery underscores the need for future research to optimize intervention strategies. The observed dissociation—where muscle mass increased but grip strength did not—suggests that Schisandra may act primarily as an anti-catabolic agent (preserving structure) rather than a potent anabolic or neurotrophic driver. Functional recovery often requires not only muscle bulk but also improvements in neuromuscular junction integrity and myofiber quality, which may not be fully addressed by Schisandra intervention alone within the experimental durations studied.

### 4.3 Limitations

Firstly, the analysis of the 11 included studies indicated substantial heterogeneity across multiple outcome measures, which could compromise the reliability of the findings. Moreover, our investigation was limited to approximately ten indicators, potentially neglecting others with smaller sample sizes. Although an ongoing meta-analysis is underway, results pertaining to these indicators remain preliminary and should be interpreted with caution. Further data are required to ascertain whether Schisandra exerts significant effects on these outcomes. The overall validity of the preclinical findings is challenged by the methodological quality of the included studies, alongside questions about the robustness and reproducibility of the data. Hence, there remains an urgent requirement for large-scale, rigorously designed studies—employing computer-generated randomization, double-blinding, and integrated pharmacokinetic monitoring—to corroborate the current findings and support future clinical translation with high-quality evidence. In addition, although structural outcomes such as muscle weight and fiber cross-sectional area showed improvement, corresponding functional outcomes (e.g., grip strength) did not reach statistical significance. This structure–function discrepancy suggests that morphological preservation alone may be insufficient to ensure functional recovery. Importantly, the lack of significant improvement in functional outcomes (e.g., grip strength) and the inconclusive dose-response relationships observed in our meta-regression analyses represent major caveats. These negative findings imply that Schisandra may predominantly exert anti-catabolic or structure-preserving effects rather than promoting true functional or anabolic recovery. Furthermore, the absence of a clear dose-dependency across studies limits our ability to propose a standardized therapeutic window for clinical use. Consequently, the translational potential of Schisandra for sarcopenia may be more limited than its structural benefits suggest, and the current evidence should be viewed as hypothesis-generating rather than confirmatory. Future research must integrate more rigorous functional assessments to reconcile these discrepancies.

## 5 Clinical value and future perspectives

This meta-analysis systematically quantifies the comprehensive effects of Schisandra in improving muscle atrophy in animal models, confirming that it exerts muscle-protective effects through a triple synergistic mechanism—antioxidant defense, inhibition of protein degradation pathways, and interruption of the inflammation–oxidative stress cycle. These results provide evidence for Schisandra as a candidate drug for combating muscle atrophy, particularly highlighting its clinical translation potential. As a GRAS-certified natural compound, Schisandra avoids the immunogenic, cardiovascular, and metabolic side effect risks associated with traditional sarcopenia treatments (e.g., androgens and growth hormones), making it especially suitable for complex, long-term conditions such as sarcopenia in the elderly (with a global prevalence of 10%–16% in individuals aged 60 and above) and cancer cachexia. However, current evidence has notable limitations, including high heterogeneity, methodological flaws, and insufficient functional improvement. Based on this, future research should focus on establishing standardized models and a core efficacy evaluation system, integrating muscle weight, cross-sectional area of muscle fibers, grip strength, and expression changes in key genes to form a multidimensional assessment framework. To enhance drug absorption efficiency, the development of specialized nanocarriers for delivering the active ingredients of Schisandra could also be explored. Innovative directions include developing personalized dosing regimens based on individual metabolic profiles of the drug. To accurately assess the anti-muscle atrophy effects of Schisandra, the generalizability and reliability of our findings must be corroborated by robust, large-scale preclinical investigations.

## 6 Conclusion

This study demonstrates the promise of Schisandra for muscle atrophy drug development and delivers an evidence-based assessment, thereby facilitating the clinical translation of the resulting findings. Schisandra increases muscle weight, enhances CAT activity, and shows a borderline significant improvement in the morphometric analysis of myofiber size. These effects stem from its multi-target mechanism, including significant inhibition of key muscle degradation factors MuRF1 and Atrogin1, accompanied by a decline in malondialdehyde concentrations. This highlights the effectiveness of Schisandra in protecting muscle structure through antioxidant defense and the inhibition of the ubiquitin-proteasome pathway. These findings may provide valuable insights for the development of Schisandra. However, accurately assessing the therapeutic potential of Schisandra for combating muscle atrophy requires validation of our results in robust preclinical trials with expanded sample sizes.

## Data Availability

The original contributions presented in the study are included in the article/supplementary material, further inquiries can be directed to the corresponding author.
